# Colchicum in Gout

**Published:** 1889-08

**Authors:** B. Simmons

**Affiliations:** Sydney, N. S. W.


Sydney, N. S. W. B. Simmons, M. D.

---

### COLCHICUM IN GOUT.

E. F., aged thirty-six, mother of two children, *leuco-phlegmatic* temperament, consulted me for rheumatism of the hands, which were *swollen*, the joints stiff and *powerless*, pain as if bruised. The whole of both arms was somewhat affected, the chief distress being in the hands. She was unable to brush her own hair, *not so much from the pain which this movement occasioned as from the extreme weakness and powerlessness of the parts affected.* She was unable to perform her usual domestic duties and had sought relief in vain. Colch.$^{cm}$ (Fincke), one dose dry on the tongue, produced a severe aggravation, lasting several hours, followed by steady improvement. After one dose of Colch. she would remain well for weeks or months, but the same remedy always helped her when repeated. Colch. has enjoyed a reputation as a remedy for gout for many years, but as followers of Hahnemann we are not content with a medicine and a name. It is for us to discover to which form of gout Colch. is homœopathic. In the *Materia Medica* we find symptoms of this kind—"laming pain in the arms which makes it impossible to hold the lightest thing;" "œdematous swelling of the hands." It is precisely in cases presenting these characteristic symptoms that we shall find Colch. curative.

Colch. seems to *paralyze and render powerless* the parts affected, and when we find with this condition *œdematous swelling* occurring in a *leuco-phlegmatic constitution,* we may expect a cure by the administration of this drug. I may here remark that the patient above referred to subsequently fell into the hands of a homœopathic physician who habitually gives alternate doses of the mother tincture and lowest potencies, and I was informed by her relatives that the medicines never relieved her rheumatism when administered in this way.

